# Successful treatment of severe cerebral malaria with artificial liver blood purification technology: A case report

**DOI:** 10.1097/MD.0000000000047528

**Published:** 2026-01-30

**Authors:** Chaoyue Fan, Yaping Dai, Hejuan Du, Tingting Su, Xiaoye Guo, Zhihan Yan, Huijing Fang, Yueping Yao, Xueshi Zhou

**Affiliations:** a Wuxi Medical College of Jiangnan University, Wuxi, China; b Department of Hepatology, The Fifth People’s Hospital of Wuxi, Jiangnan University, Wuxi, China.

**Keywords:** artificial liver blood purification, blood purification, case report, continuous renal replacement therapy, multiple organ failure, plasma exchange, severe cerebral malaria

## Abstract

**Rationale:**

*Plasmodium falciparum* infection can lead to acute thrombocytopenia, severe hemolytic anemia, and acute liver and kidney failure, among which the fatality rate of cerebral malaria is as high as 20% to 30%. Continuous bedside blood purification technology is an important intervention measure for severe malaria. The artificial liver blood purification technology, with its multimode clearance advantage, is widely used in the treatment of critical conditions such as liver failure, sepsis, and novel coronavirus infection. We described a case of severe cerebral malaria complicated with severe liver and kidney injury, cerebral edema and heart failure. The patient was cured after treatment with artificial liver blood purification technology. A literature review was also conducted.

**Patient concerns:**

A 43-year-old male patient was admitted to our hospital due to fever and confusion. The patient had frequently traveled to Nigeria on business in the past 10 years. Three days ago, he developed high fever and confusion. Blood smear examination revealed infection with *Plasmodium falciparum*. He also suffered from acute liver and kidney function impairment, severe thrombocytopenia, coagulation dysfunction, cerebral edema, and anuria.

**Diagnoses:**

Peripheral blood smear, for the diagnosis of malignant malaria parasite infection.

**Interventions:**

The patient received treatments such as artemether for antimalarial parasites, artificial liver blood purification, and ICU support.

**Outcomes:**

Through continuous renal replacement therapy combined with artificial liver blood purification technology for fluid management, immune complex clearance, and correction of water, electrolyte and acid–base disorders, the patient was successfully treated.

**Lessons:**

The integration of artificial liver blood purification with continuous renal replacement therapy may serve as an effective rescue therapy for severe malaria with multi-organ failure, potentially by mitigating the systemic inflammatory response and supporting organ recovery. This case highlights the potential of combined extracorporeal support in managing critical tropical infections.

## 1. Introduction

In 2021, the World Health Organization certified China as malaria-free.^[1]^ However, imported cases, particularly of *Plasmodium falciparum*, continue to pose a significant health threat and can lead to severe and fatal complications, including cerebral malaria and multi-organ failure.^[2]^ The management of these critical cases often requires intensive care support beyond antimalarial drugs. This case report details the successful use of combined artificial liver blood purification and continuous renal replacement therapy (CRRT) in managing a patient with severe cerebral malaria and multi-organ dysfunction, providing a valuable perspective on adjunctive treatment strategies in a post-elimination setting.

## 2. Case report

**Figure 1.**
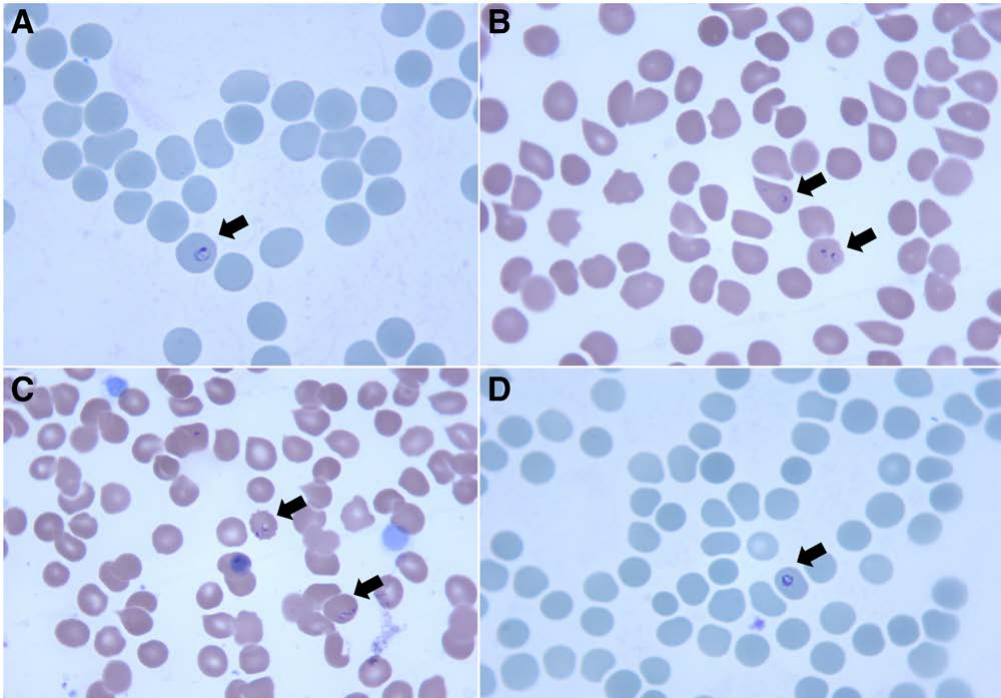
Initial thin blood smear microscopy of the patient at disease onset (1000× magnification). The black arrow indicates ring forms (early trophozoites), consistent with the microscopic characteristics of *Plasmodium falciparum*.

**Figure 2.**
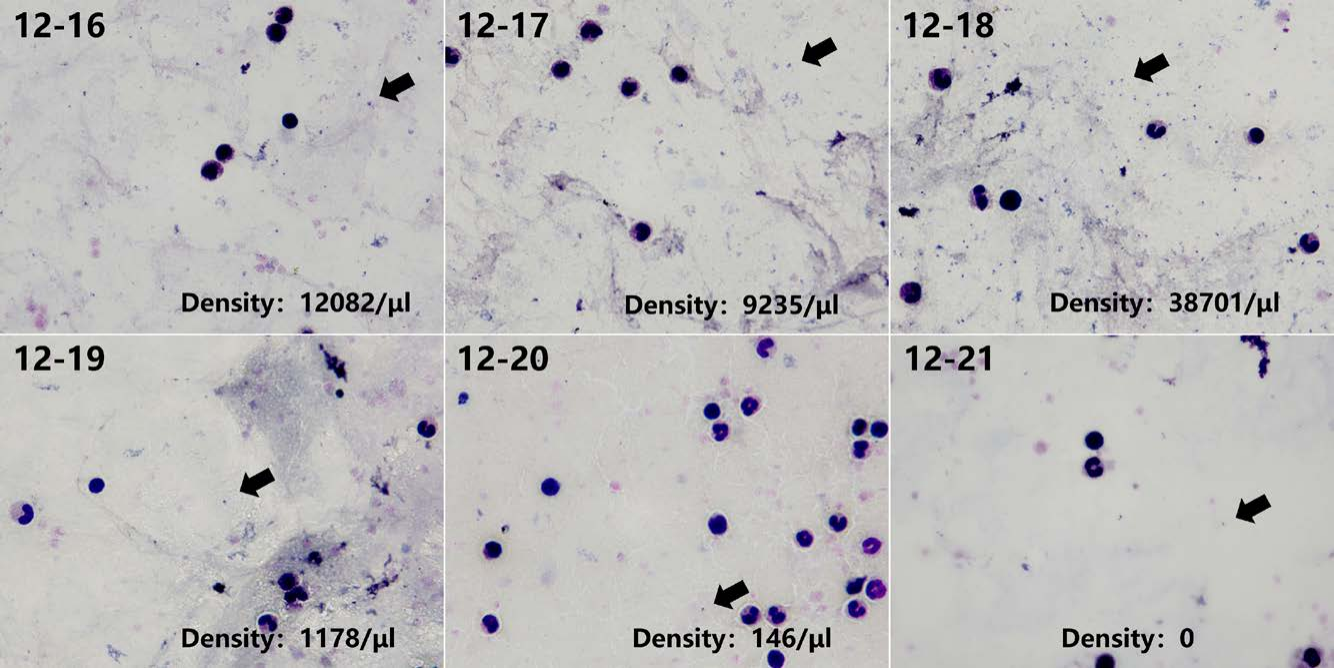
Thick blood smear microscopy during the patient’s acute illness (1000× magnification). Black arrows indicate malaria parasites, demonstrating progressive parasite density reduction following antimalarial treatment.

**Figure 3.**
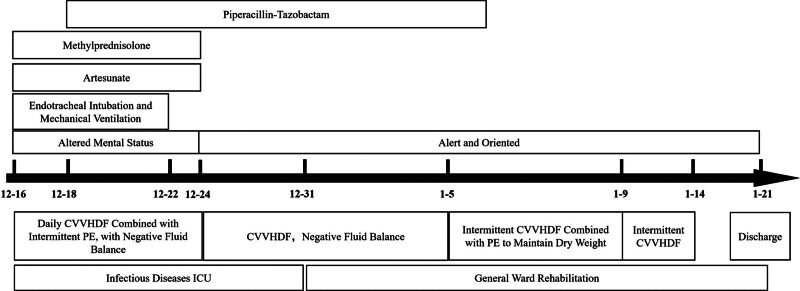
Treatment timeline of the patient. From December 16 to December 24, rapid parasite clearance was achieved following artesunate therapy, accompanied by gradual restoration of consciousness and successful extubation. After a prolonged anuric phase requiring continuous renal replacement therapy (CRRT), diuresis progressively resumed with concurrent renal function recovery. During this period, refractory anemia developed and was managed with intermittent plasma exchange, effectively correcting immune-mediated hemolysis.

The patient is a 43-year-old male who was admitted on December 16, 2024 with “fever for 3 days and unconsciousness for 5 hours.” He had frequently traveled to Nigeria on business over the past decade, with his most recent return to China half a month prior. Three days before admission, he developed remittent fever (peak temperature 39°C) accompanied by lethargy. Five hours preadmission, he experienced sudden loss of consciousness with no response to stimuli, and was transported to our emergency department via ambulance (120 emergency system). Vital signs on admission: temperature 36.6°C, pulse 122 beats/min, respiratory rate 18 breaths/min, blood pressure 146/86 mm Hg. Physical examination revealed: comatose state (Glasgow coma scale 3), severe icterus of skin and mucous membranes, scleral icterus with mild chemosis, pupils isocoric (3 mm in diameter) with sluggish light reflex, cardiopulmonary auscultation: sinus tachycardia (122 beats/min), clear lung sounds bilaterally, soft abdomen without guarding, no edema in lower extremities, no pathological reflexes elicited. Key laboratory results on admission and during treatment are summarized in Table 1. Emergency blood smear examination identified *Plasmodium falciparum* (Fig. 1), with a parasite density of 10,000/μL on December 16 (Fig. 2). CT scans of the head, chest, and abdomen showed no abnormalities. Diagnosis: *Plasmodium falciparum* infection (cerebral malaria), multiple organ dysfunction syndrome comprising: acute liver injury, acute kidney injury (kidney disease improving global outcomes stage 3), severe thrombocytopenia, metabolic acidosis (lactic type). Administer low-molecular-weight heparin 4000 IU subcutaneously once daily for microthrombosis prophylaxis. Intravenous high-dose vitamin C (6 g/d) is given to improve microcirculatory dysfunction, combined with methylprednisolone (40 mg twice daily) intravenous infusion to control systemic inflammatory response (treatment course: 7 days). Concurrently implement enteral nutritional support while maintaining capillary blood glucose within 8 to 11 mmol/L through insulin titration. For intracranial hypertension, administer a combined regimen of glycerin fructose sodium chloride and human albumin for intensive dehydration and intracranial pressure reduction therapy. The patient presented with persistent anuria (24-hour urine output < 100 mL), accompanied by severe chemosis and optic nerve sheath edema, with bedside ultrasound confirming pulmonary edema. CRRT was maintained for 10 hours daily (net ultrafiltration volume >1000 mL/d), supplemented with 3 sessions of plasma exchange during the first treatment week (exchange volume 2000 mL per session). On December 21st, after the malaria parasite blood smear turned negative, artesunate was continued for a 3-day consolidation course, followed by a 2-day regimen of dihydroartemisinin–piperaquine tablets administered via nasogastric tube (see Fig. 2). Dynamic platelet monitoring revealed a nadir of 17 × 10^9^/L, prompting prophylactic platelet transfusion to ensure CRRT safety. For severe anemia (hemoglobin nadir 56 g/L), fractionated suspended red blood cell transfusions were administered. After successful extubation on December 23rd, the patient was transitioned to high-flow humidified oxygen therapy, though somnolence persisted. Chest imaging demonstrated pulmonary inflammation, prompting empirical therapy with piperacillin–tazobactam (4.5 g every 8 hours). Bronchoalveolar lavage fluid cultures showed no pathogenic organisms. Continuous central venous pressure monitoring guided CRRT ultrafiltration volume. The patient developed refractory anemia persisting after malaria parasite clearance, which was attributed to artesunate-induced immune hemolytic anemia. Anemia improved following 2 sessions of plasma exchange for immune complex removal. After dialysis access revision (removal of femoral hemodialysis catheter with transition to internal jugular vein access) on December 31st, the patient was transferred to the general ward and transitioned to hemodiafiltration 3 times weekly. On January 9th, the patient’s daily urine output was approximately 100 mL. The treatment regimen during hospitalization is summarized in Figure 3.

**Table 1:** Dynamic changes of key laboratory indicators during treatment.

Variable	On admission (December 16)	Day 11 (December 27)	Day 24 (January 9)	At discharge (January 21)
Total bilirubin (µmol/L)	291.1	37.6	18.3	18.3
Direct bilirubin (µmol/L)	183.3	25.7	6	6
ALT (U/L)	117	–	10	10
Creatinine (µmol/L)	227.8	1269	651.3	197
Urea (mmol/L)	14.1	46.9	12.4	10.2
Platelets (×10^9^/L)	20	17	197	210
Hemoglobin (g/L)	127	56	89	95
pH	7.33	7.39	7.41	7.42
Arterial lactate (mmol/L)	7.1	2.3	1.5	1.2
Glasgow coma scale (GCS)	3	–	–	15

## 3. Discussion

With the deepening of Sino-African economic and trade exchanges, the number of imported malaria cases in China has shown an upward trend in recent years.^[3,4]^ Unlike indigenous malaria, imported cases often present with severe manifestations due to delayed diagnosis and lack of immunity in the local population.^[5]^ This patient had a long-term travel history to Nigeria but did not receive preventive antimalarial treatment, leading to rapid progression to severe cerebral malaria and multiple organ dysfunction syndrome. It is emphasized that for patients with a history of travel to malaria-endemic areas, timely malaria parasite detection (combined thick–thin blood film microscopy) is crucial for early diagnosis,^[6]^ which is consistent with the WHO severe malaria management guidelines.^[7]^

*Plasmodium falciparum* infection activates the host immune system, leading to the release of a large number of inflammatory mediators (IL-6, IL-8, TNF-α, etc) and the formation of immune complexes.^[8,9]^ These substances damage vascular endothelial cells, induce microthrombosis, and ultimately cause multi-organ dysfunction.^[10,11]^ The combined application of artificial liver blood purification and CRRT exerts a synergistic therapeutic effect:

1. Plasma exchange can directly remove plasmodium parasites, macromolecular immune complexes, and pro-inflammatory cytokines,^[12,13]^ which is particularly important for relieving immune-mediated hemolysis and cerebral edema.
2. CRRT maintains fluid-electrolyte and acid–base balance, reduces the accumulation of small-to-medium molecular toxins, and improves renal perfusion.^[14,15]^

**Figure 4.**
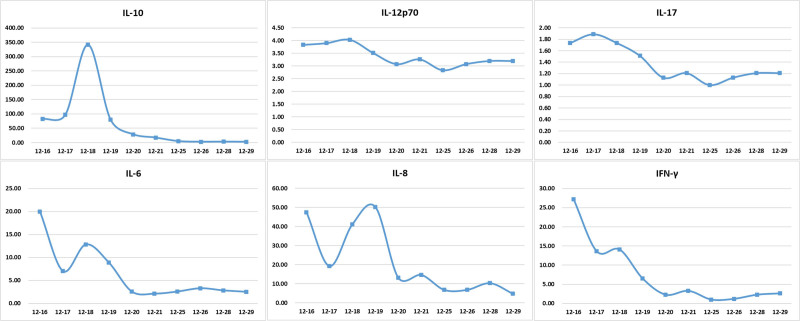
Trend analysis of peripheral blood inflammatory cytokine levels during disease progression. IL-10, IL-12P70, IL-17, IL-6, IL-8, and IFNγ showed significant elevation during the acute phase, followed by a progressive decline, demonstrating the intense inflammatory cascade triggered by *Plasmodium falciparum* infection.

In this case, the patient’s inflammatory cytokines (IL-10, IL-12P70, IL-17, etc) showed a significant downward trend after combined therapy (Fig. 4), confirming the anti-inflammatory effect of extracorporeal support. This is consistent with the findings of Meremo et al^[16]^ and Boushab et al,^[17]^ who reported that continuous blood purification can improve the prognosis of severe malaria with acute kidney injury (AKI).

### 3.1. Management of key complications

1. *AKI*: AKI is a common complication of severe *Plasmodium falciparum* infection, with a pathogenesis involving erythrocyte deformation obstruction, inflammatory response, and hemodynamic disturbance.^[18]^ This patient developed anuric AKI (kidney disease improving global outcomes stage 3) on admission, and CRRT was initiated early to prevent the progression of acute kidney disease to chronic kidney disease.^[19]^ The patient’s renal function recovered after 35 days of extracorporeal support, which supports the view that proactive CRRT can reverse renal dysfunction in malaria-associated AKI.^[20]^
2. *Immune hemolytic anemia*: Artesunate-induced autoimmune hemolytic anemia is a rare adverse reaction, characterized by persistent hemoglobin decline after parasite clearance.^[12]^ In this case, plasma exchange was used to remove autoantibodies and immune complexes, and the patient’s anemia improved significantly, which is consistent with the therapeutic experience reported by Silberstein et al.

This is a single case report, and the therapeutic effect needs to be verified by large-sample clinical studies. In addition, the long-term prognosis of the patient requires further follow-up.

## 4. Conclusion

For patients with severe cerebral malaria complicated by multi-organ failure, early diagnosis combined with artificial liver blood purification ± CRRT extracorporeal support therapy can effectively clear toxins and inflammatory mediators, protect organ function, and improve the cure rate. This combined strategy provides a valuable clinical reference for the management of imported severe *Plasmodium falciparum* malaria in post-elimination China. Clinicians should pay attention to the travel history of patients, strengthen early pathogen detection, and actively use comprehensive intervention measures to improve the prognosis of severe cases.

## Author contributions

**Conceptualization:** Hejuan Du.

**Data curation:** Yaping Dai.

**Formal analysis:** Xiaoye Guo.

**Resources:** Tingting Su.

**Software:** Huijing Fang, Yueping Yao.

**Visualization:** Zhihan Yan.

**Writing – original draft:** Chaoyue Fan.

**Writing – review & editing:** Xueshi Zhou.

## References

[1] Venkatesan PT. 2023 WHO world malaria report. Lancet Microbe. 2024;5:e214. 38309283 10.1016/S2666-5247(24)00016-8

[2] Lai S Sun J Ruktanonchai NW. Changing epidemiology and challenges of malaria in China towards elimination. Malar J. 2019;18:107. 30922301 10.1186/s12936-019-2736-8 PMC6440015

[3] Mzumara G Leopold S Marsh K Dondorp A Ohuma EO Mukaka M. Identifying prognostic factors of severe metabolic acidosis and uraemia in African children with severe falciparum malaria: a secondary analysis of a randomized trial. Malar J. 2021;20:282. 34172046 10.1186/s12936-021-03785-0 PMC8234663

[4] Zhang L Yi BY Zhou SS Xia ZG Yin JH. Epidemiological characteristics of Plasmodium malariae malaria in China: a malaria that should not be neglected post elimination. Infect Dis Poverty. 2023;12:101. 37986018 10.1186/s40249-023-01156-2 PMC10658989

[5] ZChoy B Bristowe H Khozoee B Lampejo T. Increased imported severe *Plasmodium falciparum* malaria involving hyperparasitaemia (≥10%) in a UK hospital following relaxation of COVID-19 restrictions compared to the pre-pandemic period. J Travel Med. 2022;29:taac116. 36209409 10.1093/jtm/taac116 PMC9619718

[6] Dong Y Deng Y Xu Y. Analysis of initial laboratory diagnosis of malaria and its accuracy compared with re-testing from 2013 to 2018 in Yunnan Province, China. Malar J. 2020;19:409. 33183296 10.1186/s12936-020-03477-1 PMC7664069

[7] Deng Q Wu Y Chen L. Compliance with WHO malaria treatment guidelines and clinical outcomes in China. Malar J. 2024;23:189.38880891

[8] Kaur C Pramanik A Kumari K. Renal detection of *Plasmodium falciparum*, *Plasmodium vivax* and *Plasmodium knowlesi* in malaria associated acute kidney injury: a retrospective case-control study. BMC Res Notes. 2020;13:37. 31959229 10.1186/s13104-020-4900-1 PMC6971858

[9] Chen Y Zhou H Yang C. Mechanisms of artificial liver blood purification in regulating systemic inflammatory response. Hepatobiliary Pancreat Dis Int. 2024;23:168–73.

[10] Vogetseder A Ospelt C Reindl M Schober M Schmutzhard E. Time course of coagulation parameters, cytokines and adhesion molecules in *Plasmodium falciparum* malaria. Trop Med Int Health. 2004;9:767–73. 15228486 10.1111/j.1365-3156.2004.01265.x

[11] Gao F Wang J Li S. Inflammatory mediators in severe malaria: a prospective cohort study. Cytokine. 2023;166:156289.

[12] Silberstein LE Berkman EM. Plasma exchange in autoimmune hemolytic anemia (AIHA). J Clin Apher. 1983;1:238–42. 6546059 10.1002/jca.2920010407

[13] Liu Z He C Zhang Y. Plasma exchange for immune-mediated diseases: an update on clinical applications. Transfus Apher Sci. 2023;62:103456.

[14] Xu J Sun Y Zhang W. The efficacy and safety of continuous blood purification in neonates with septic shock and acute kidney injury: a two-center retrospective study. Eur J Pediatr. 2024;183:689–96. 37971515 10.1007/s00431-023-05336-y

[15] Zou F Tang X Lei X Cao F Luo J Liu S. Treatment efficacy of continuous renal replacement on symptoms, inflammatory mediators, and coagulation function in patients with sepsis-associated acute kidney injury. Arch Esp Urol. 2022;75:746–52. 36472056 10.56434/j.arch.esp.urol.20227509.109

[16] Meremo AJ Kilonzo SB Munisi D. Acute renal failure in a Caucasian traveler with severe malaria: a case report. Clin Case Rep. 2014;2:82–5. 25356255 10.1002/ccr3.65 PMC4184599

[17] Boushab BM Fall-Malick FZ Savadogo M Basco LK. Acute kidney injury in a shepherd with severe malaria: a case report. Int J Nephrol Renovasc Dis. 2016;9:249–51. 27785088 10.2147/IJNRD.S116377 PMC5066854

[18] House AA Ronco C. Extracorporeal blood purification in sepsis and sepsis-related acute kidney injury. Blood Purif. 2008;26:30–5. 18182792 10.1159/000110560

[19] James MT Levey AS Tonelli M. Incidence and prognosis of acute kidney diseases and disorders using an integrated approach to laboratory measurements in a universal health care system. JAMA Netw Open. 2019;2:e191795. 30951162 10.1001/jamanetworkopen.2019.1795 PMC6450331

[20] Yang L Zhang M Liu J. Artesunate-induced autoimmune hemolytic anemia: a case series and literature review. Int J Infect Dis. 2022;126:89–94.

